# Effective SMOTE boost with deep learning for IDC identification in whole-slide images

**DOI:** 10.1371/journal.pone.0329078

**Published:** 2025-09-03

**Authors:** Mudhafar Jalil Jassim Ghrabat, Arkan A. Ghaib, Auhood Al-Hossenat, Zaid Ameen Abduljabbar, Vincent Omollo Nyangaresi, Junchao Ma, Abdulla J. Y. Aldarwish, Iman Qays Abduljaleel, Dhafer G. Honi, Husam A. Neamah

**Affiliations:** 1 University of Information Technology and Communications (UOITC), Baghdad, Iraq; 2 Computer Science Department, Al-Turath University, Baghdad, Iraq; 3 Information Technology Department, Management Technical College, Southern Technical University, Basrah, Iraq; 4 Department of Computer Science, College of Education for Women, University of Baghdad, Baghdad, Iraq; 5 Department of Computer Science, College of Education for Pure Sciences, University of Basrah, Basrah, Iraq; 6 College of Big Data and Internet, Shenzhen Technology University, Shenzhen, China; 7 Department of Business Management, Al-Imam University College, Balad, Iraq; 8 Department of Computer Science and Software Engineering, Jaramogi Oginga Odinga University of Science & Technology, Bondo, Kenya; 9 Department of Applied Electronics, Saveetha School of Engineering, SIMATS, Chennai, Tamil Nadu, India; 10 Department of IT, University of Debrecen, Debrecen, Hungary; 11 Department of Electrical Engineering and Mechatronics, Faculty of Engineering, University of Debrecen, Debrecen, Hungary; Kafkas University: Kafkas Universitesi, TÜRKIYE

## Abstract

Breast cancer is highlighted in recent research as one of the most prevalent types of cancer. Timely identification is essential for enhancing patient results and decreasing fatality rates. Utilizing computer-assisted detection and diagnosis early on may greatly improve the chances of recovery by accurately predicting outcomes and developing suitable treatment plans. Grading breast cancer properly, especially evaluating nuclear atypia, is difficult owing to faults and inconsistencies in slide preparation and the intricate nature of tissue patterns. This work explores the capability of deep learning to extract characteristics from histopathology photos of breast cancer. The research introduces a new method called SMOTE-based Convolutional Neural Network (CNN) technology to detect areas impacted by Invasive Ductal Carcinoma (IDC) in whole slide pictures. The trials used a dataset of 162 individuals with IDC, split into training (113 photos) and testing (49 images) groups. Every model was subjected to individual testing. The SMO_CNN model we developed demonstrated exceptional testing and training accuracies of 98.95% and 99.20% respectively, surpassing CNN, VGG19, and ResNet50 models. The results highlight the effectiveness of the created model in properly detecting IDC-affected tissue areas, showing great promise for improving breast cancer diagnosis and treatment planning. We surpassing other models as such, CNN, VGG19, ResNet50.

## 1. Introduction

Cancer is a huge worldwide health issue since it impacts people’s lives. Breast Cancer (BC) incidence and mortality have increased in recent years. Breast cancer refers to cancer that starts in the breast tissue [1]. Breast cancer is a disease that has devastating effects on the lives of 25- to 50-year-old women. For increased survival among patients, they must be diagnosed early and accurately [2]. A breast cancer diagnosis dependent on histological images faces three key problems, thus, the analysis made in this paper, it is evident that breast cancer diagnosis using histological images has three major challenges. First of all, it is necessary to mention that tumor tissue variant is not uniform that makes the diagnosis difficult due to the fact that even in the given tumor there may be regions that differ in the characteristics. Secondly, subjectivity and issue of variability of pathologists can be explained in the following way that because of different training and experience different pathologists will interpret the same images in different manners. Finally, a poorly stained sample or low power microscopy means the features in the tissue cannot be made out properly and hence diagnosis is affected [3]. First, experienced histopathologists are limited, particularly in developing countries and small hospitals.

Moreover, the diagnosis made by a histopathologist is based only on their experience and judgment, with no basis. Histopathologists are only responsible for the accuracy of diagnosis. Throughout the age of big data, Breast cancer detection using histological images has been time-consuming and labor-intensive, making it an inefficient approach in current times even in this era of big data, breast cancer detection in histopathology is still claimed to be time-consuming based on recent studies. The fact that tumor tissue is heterogeneous, that there is a degree of subjectivity in the pathologists’ interpretation of the slides, and that AI methods require large sets of high-quality annotated data are still the major challenges that even remarkable development of computational power and availability of the data cannot eliminate [4]. Biomedical scientists in the profession of pathologists analyze sections of tissue, cellular tissues and fluids, applying the scientific method with a range of biomarkers to evaluate various ailments particularly in the cancer field. One of the such clinical entity is tumor budding, which is histopathological feature that is defined as single cells, or clusters of cells comprised not more than four cells at the invasive front of the carcinomas most notably in the colorectal cancer and has been found to be associated with aggressive disease and has grant adverse prognostic implication [5]. Also important is tumor-infiltrating lymphocytes (TILs), lymphocytes that have invaded the tumor mass; TILs staging can be viewed as a sign of the body’s immunological reaction to the tumor and is prognostically significant especially in melanoma and breast cancer [6]. Immunodeficiency (IHC) is another very important tool it involves staining of tissue section with antibodies to detect special antigens present in tissues and used in identification of the type of tumor its origin and some special markers that may be potential targets of treatment. Pathologists also carry out biomarker analysis for instance, HER2 in breast cancer [7], EGFR mutations in non-small cell lung cancer. However, more scientific progress in the subject has been conducted progressively refining the pathology as a field with mutual developments in the diseases’ mechanisms, biomarkers appearance, and technical improvements in the known methods. The processes of digital pathology and computational image analysis should be developed in such a manner where integration of these methodologies actively utilizes and expands the basis originated from this science [8]. Also, progression in digital pathology and use of artificial intelligence in pathology are implemented in current pathology practice, providing quicker and more precise analysis of images as well as decision making. The dynamic nature of pathology reinforces the crucial role that pathologists play in modern medicine. Their expertise and use of cutting-edge tests not only result in accurate diagnoses, but also enhance patient care by applying a scientific foundation for treatment choices [9]. Given these limitations, histopathologists’ efforts must be reduced to establish an efficient and objective method of disease detection.

IDC accounts for about 80% of all cases of breast cancer. Another kind of breast cancer, invasive ductal carcinoma starts in the breast duct and spreads to other areas of the breast. This disease exhibits a great tendency for metastasis, it might spread through Lymph nodes and blood vessels. Therefore, it is critical that the sickness be identified early so that the patient or customer may get the proper therapy. Most of the time the IDC is discovered when; Mammography reveals opaqueness and a suspicious mass or deposits of micro calcification. If there is clinically or radiographically a suspicious lesion, then a biopsy is carried out to establish its nature. As for the imaging education in the assessment, help can also come from ultrasonography and MRI. The common treatment plans of IDC include surgery, radiation, chemotherapy, hormonal therapy, and targeted therapy. The possible intervention options depend on several factors relating to the cancer, such as the stage and characteristics, the condition of the patient, and other possibilities. Forecast: It depends with the stage of the diagnosis; other factors include hormone receptor status as well as HER2. Regarding IDC particularly, it clearly illustrated that cosmopolitan early-stage rates are higher as compared to the rates of the delayed stage. DL has been found useful when it comes to diagnosing multiple clinical diseases inclusive of IDC. However, despite the considerable attention they have received, these methods still have several limitations and difficulties: Nevertheless, as the literature review demonstrates, they have several drawbacks and difficulties even though they have attracted a lot of attention: Quality and quantity of data: On the topic chosen, it is possible to distinguish two primary points of focus: the quality of collected data and the amount of data obtained. This is the reason that for the training of these deep learning models they need clean annotated medical pictures. But in order to obtain more numerous, but better quality IDF photos, this may become a problem because it is necessary to obtain permission from the patient not to mention the critical time it takes to annotate by qualified physicians.

Some features of the data variation may lead to the inability to apply data from different cohorts, demographics, and clinical settings for AI model use. Such differences may be due to differences in imaging equipment, the methods used to collect images, and the studied patients. Scalability is another problem in regard to the efficient unification of several detached collections containing photos into extensive databases [9].

Machine Learning (ML) has been utilized to detect images, recognize objects, and classify text. Using CAD technology, ML has been effectively used in the early detection of breast cancer [10]. Histopathology image classifications via deep learning (DL) approaches are common, including artificial feature extraction and the classical machine learning approach. Machine Learning and artificial feature extraction for histopathological image classification require human feature creation but do not require high-performance components and have benefits in computation time. Considering that these methods can solve issues of classical feature extraction [11] in computer vision, biomedical science, and many other domains. Recently, transfer learning has remarkably arisen for scenarios when big, annotated datasets are still unavailable [12]. Pretrained networks in transfer learning are also trained using ImageNet, a large-scale dataset [13]. The generalization performance of pre-trained models for applications with fewer classes is among the worst, and over-parameterized models result in overfitting [14–17]. Depending on deep learning, particularly CNN, histopathological image classification frequently needs the highest number of labeled training samples; however, obtaining labeled data is problematic. Histopathologists who are well-known for labeling lesions report that this process is hard and time-consuming [18,19]. Pre-trained models outperform untrained methods and are now the best option for training models on additional datasets rather than training a model from scratch [20–22]. However, these pre-trained models cannot be customized according to need.

It is clear from the issue description that analyzing the histopathological pictures is a tedious and time-consuming procedure that requires expert assistance in order to discover any illness or condition. Moreover, the specialization of pathologists who would perform the analysis can also affect the overall assessment result. Automated histopathological image analysis refers to using computers to analyze images in order to diagnose as well as forecast individuals with breast cancer effectively. The challenges that need to be surmounted to design tools for this research are as follows. First, histopathological pictures of breast cancer contain fine details from geometric features and intricate textures of the tissues. Classification can turn out to be very rigorous in the case of multiple classes, when there are variations possible within a class and interchangeability between classes. The second problem that has to be solved is the limitation of the feature extraction for histopathology imaging of breast cancer. Moreover, prior exposure to data is needed when selecting a number of essential variables, which impacts the extraction effectiveness but increases the amount of computations that the machine must perform. The degree of and kind of histopathological imaging that may be retrievable in the final result may be low-level and unrepresentative. This condition may result to poor classification results for the final model. However, when the numerical of the categories is sufficiently small, these kinds of pre-trained models involve poor generalization ability, resulting in over-parameterized networks, which is unfavorable [23].

However, despite the great attempt in addressing this problem, the exact solution has not been arrived at. The previous research outlines the following research gaps or disadvantages:

1. Accurate breast cancer diagnosis employing automatic methods remains a major challenge in the research.
2. The difficulty is aggravated by almost all available datasets being unbalanced. In other words, the number of cases of one class vastly outnumbers those of all the others.
3. Unfortunately, pre-trained models were developed using large standard datasets like ImageNet, but these datasets lacked labeled pictures pertinent to breast cancer, resulting in low performances.
4. Pathologists also find identifying malignancy areas in Whole-Slide Imaging (WSI) difficult.
5. All previous models were pre-trained CNN models and cannot be customized.

The fundamental goal of this research is to provide a solution based on the SMOTE-based CNN model for overcoming the challenges mentioned above. The study aims to build an efficient IDC classifier using only a customized model’s significant feature variables. To address this important issue, we present this work’s significant contributions. A framework was created utilizing the CNN model to address the problem of limited generalization capability in fast decision-making while recognizing histopathology images. Then, produce a complete literature review on breast cancer using various methodologies. Second, a SMOTE method was presented for dealing with data imbalances. Then, we describe a histopathological image identification system based on deep learning. Third, a huge dataset was used to increase the ability of IDC histopathology image categorization.

Finally, a simple strategy is designed to adapt, has good accuracy, and can identify breast cancer in positive and negative classes. This study’s main goal is to improve CNNs’ performance while dealing with unbalanced datasets. The first objective proposes a novel approach leveraging. The application of CNN in various fields has shown promising results, this imbalance can significantly impair a model’s ability to accurately forecast minority class instances. The SMOTE has been identified as a potential solution to this issue, yet its integration with CNN training processes is not well-explored. Addressing these gaps is vital for enhancing the robustness and accuracy of CNN models, particularly in scenarios where data imbalance is prevalent. My research is motivated by the need to bridge these gaps, focusing on the effective integration of SMOTE with CNN training to develop models that are both balanced and highly accurate.

The portions of this paper are as follows: The literature study discussed in the next part outlines current ML and DL models and focuses on whole-slide pictures for breast cancer screening. The research’s problem statement and motivation were covered in **Section** **3**. The suggested technique is then methodically described in **Section 4**, which also includes information on the algorithm and flowchart. The experimental results for the suggested approach and a comparison with pre-trained models are shown in **Section 5**. Finally, **Section 6** provides a summary of the findings of this study and suggests potential avenues for further investigation.

## 2. Literature review

Significant research has been conducted in the decades of years on using machine learning in disease prediction, especially in breast cancer detection. For more than 40 years, researchers have been studying breast cancer detection using image analysis, and they have made tremendous progress. Their research is classified into classical ML methods and DL models based on their methodology. This section provides a comprehensive evaluation of new improvements in breast cancer detection. Various prediction models are available in the literature.

Chen (2022), develop an AAU-net (adaptive attention U-net) with the purpose of accurately and autonomously segmenting breast lesions from ultrasound pictures. The authors propose the implementation of a novel and versatile attention module called HAAM, as well as the replacement of the traditional convolution operation with a CSAB and a spatial CSAB. The hybrid adaptive attention module may gather more information from various receptive fields than a conventional convolution operation. The network may be guided by the HAAM module to dynamically choose a more robust channel and space representation to efficiently manage the segmentation of increasingly complex breast lesions. Extensive studies performed on three publicly available breast ultrasound datasets reveal that our method outperforms modern DL segmentation methods in properly segmenting breast lesions [24].

In this study Chen (2021), the authors proposed a new architecture for CNNs termed “multi-scale fusion of structural features and detailed features (SDFNet)” to segment kidneys. The S-Net, D-Net, and MCBlock collaborate to extract structural characteristics, gather texture data, and combine features. The S-Net team created boundary detection (BD) module to get a more thorough understanding of the structural properties of the kidney. Furthermore, this study devised a systematic training approach to improve the SDFNet’s ability to generalize. We conducted a thorough evaluation of the recommended technique’s precision and compared it to several methodologies, using six quantitative measures on the same renal ultrasound dataset. The findings indicate that the recommended strategy has the greatest overall performance in segmenting renal ultrasound pictures [25].

An iterative up-sampling optimization strategy was devised in [26] to decrease the complexity of the network’s design. We evaluate several medical picture segmentation methods using a comparable KUS dataset and seven quantitative metrics. The performance of our approach is as follows: 89.95% on Jaccard, 94.59% on Dice, 94.47% on Accuracy, 95.07% on Recall, 0.3006 on Average Symmetric Surface Distance (ASSD), and 0.9703 on AUC. The experimental findings demonstrate the superiority of the suggested strategy over the previously used ones for KUS picture segmentation.

This study, Chen (2023); Chen (2022) offers a novel approach to classifying breast tumours from ultrasound images using a refinement residual convolutional network. An important part of the network is SegNet, which has a deep supervision module. Other important parts include a residual network for missed detection and one for false detection. The network’s segmentation performance was compared to various state-of-the-art segmentation approaches in our investigation. This analysis was based on five quantitative characteristics and used two publicly available breast datasets. Our experimental findings unequivocally show that our technique achieves outstanding segmentation, indicating its potential usefulness in segmenting breast tumors [27,28].

The study, Chen (2022) used the U-net, BAGNet, and RFNet models to develop a new cascaded CNN for accurately identifying and segmenting lesions in breast ultrasound images. We conducted a comparison of the network’s segmentation effectiveness with other advanced segmentation techniques using six traditional evaluation measures, utilizing the publicly accessible breast ultrasound dataset (BUSIS). Our technique outperforms others on six distinct factors. Furthermore, the p-values indicate that our technique exhibits a substantial divergence from the other approaches. The experimental results indicate that our solution surpasses other approaches in terms of segmentation accuracy [29].

Contrast-enhanced digital mammogram (CEDM) is a new technique that combines a limited-energy (LE) picture, similar to FFDM, with a reconstructed image that highlights tumor neoangiogenesis, similar to breast MRI scans. In their study, the authors [30] presented a telecare approach for breast cancer screening in rural Indian women using Telemammography solutions. The objective of this approach is to dynamically initiate the production of a summary based on a given collection of input test sets. The algorithm incorporates the division of the test’s images into segments and the enhancement of their contrast to enhance processing. It also includes an image analysis tool that calculates a condensed value for the input test sets. This value is then compared to a predetermined threshold to assess the likelihood of breast cancer and generate reports.

The use of the CNN deep feature fusion technique for breast detection has been examined in [31]. Convolution neural network deep features and unsupervised ELM clustering were used in the first step of the mass detection technique. They combined the previously discussed features for the second step to create a new feature set. An ELM classifier capable of differentiating between benign and malignant breast tumours was then trained using this combined collection of features. After a battery of rigorous tests, their proposed methods for mass detection and breast cancer categorisation were shown to be effective and efficient. This study’s overarching goal is to assess how well CNNs, ANNs, and MLP neural networks do in the early detection of breast cancer [32]. A unique transfer learning concept-based deep learning approach has been developed for identifying and monitoring malignancy in breast cytology images [33]. Deep learning approaches are often designed to be conflict and executed in separation. The suggested approach extracts features from an image using pre-trained CNN models, including GoogleNet, ResNet, and VGGNet. These were loaded into dense layers for the categorization of malignant from benign cells that use average pooling categorization. Tests were conducted using standard test data to evaluate the suggested approach’s effectiveness. The proposed approach outperformed all previous deep learning models with accuracy in detecting and classifying breast tumors in the cytology pictures.

Using this data, an ensemble deep-learning approach has been devised for categorizing carcinoma and non-carcinoma breast cancer histopathology images [34]. They used pre-trained VGG-16 & VGG-19 architectures to build four models. Each model, including fine-tuned and fully trained VGG16, was initially subjected to a five-fold cross-validation procedure. Upon averaging projected probability, the VGG16 fine-tuned ensemble and VGG19 performed competitively in the carcinoma class, especially compared to other algorithms. For the carcinoma classification, VGG16 and VGG19 models that have been finely tuned as an ensemble had an overall accuracy of 95.29% and a sensitivity of 97.73% for the carcinoma class. The F1 score was 95.29%. According to the results of these experiments, these learning models can also be used to automatically classify complex-natured breast tumor histopathology images, particularly the carcinoma image. A system founded on the principle of transfer learning can be used to handle the problem of extremely unbalanced classification tasks and redouble attention to histological and unevenly distributed classification tasks [23]. Researchers have improved the system’s overall performance by using the well-known VGG-19 as a baseline model and augmenting it with several new approaches. They utilized the trained data in the particular domain of histopathology images by using the ImageNet dataset as the input space. Experimental research on a huge database of 277,524 images demonstrates that the structure presented in the present study outperforms these few current structures in the published literature.

Many pre-trained models built on big benchmark datasets, including ImageNet, do not consist of labeled pictures of breast tumors, resulting in low performances. A unique approach termed double-shot transfer learning (DSTL), based on the transfer learning concept, has been described in [35,36]. DSTL is being applied to increase the performance and accuracy of pre-trained breast cancer classification models. Double-shot transfer learning fine-tunes the trainable variables (weight and bias) of one of the pre-trained models using a big dataset identical to the targeted data source. The revised models are then fine-tuned using the targeted data. Furthermore, overfitting is prevented, and insights are provided by increasing the count of X-ray images by using a combination of data augmentation approaches that include distinct variants of rotations, intensity, flips, and contrasts. The suggested method remarkably improves pre-trained models’ classification performance and accuracy, making them better suited for diagnostic imaging.

Utilized transfer learning, a DL approach that involves adapting a pre-trained model from one task to a related new task. TL was used to develop a sophisticated DL model specifically designed for automated breast cancer diagnosis and detection used an 80–20 cross-validation method to train and assess the model. This method entails randomly dividing the dataset into two segments: 80% for training the model and 20% for evaluating its performance. By iterating this procedure many times (usually ten) with distinct partitions each time, the model’s resilience and generalization may be evaluated. Moreover, the researchers created deep-learning architectures tailored to particular problems. These architectural designs are customized to tackle the particular problems and features of the breast cancer detection process. This modification entails creating neural network layers, activation functions, and optimization algorithms specifically tailored for detecting cancer-suspicious regions in breast pictures. The integration of transfer learning, cross-validation, and problem-specific deep-learning architectures offers a strong foundation for creating and assessing DL models for automated breastcancer diagnosis and detection [37].

Our study analyzed conventional ML techniques and DL models in cancer research. Diagnostic images for cancer diagnosis, diagnostic images for diagnostic testing, and automatic analysis in cancer care are all included in this paper, which offers a detailed comparison of prior research efforts using ML-based and DL-based methodologies. DL algorithms are the subject of this investigation because they routinely outperform machine learning alternatives. Deep learning was used as the foundation for many of the methodologies presented in the numerous articles, and these methods were found to be highly predictive. The paper describes cancer complications and treatments, AI-based strategy cancer classification, DL’s contribution to cancer research, the barriers to cancer forecasting utilizing algorithmic training, ongoing investigations, and difficulties associated with cancer studies using deep learning-based methods.

Many experts consider deep learning (DL) to be effective for IDC diagnosis despite ongoing diagnosis obstacles. The large complexity and multiformity of medical image information creates challenges for training processes. The process of supervised learning demands dataset annotation with expert labels while only medical professionals can provide these labels because of the time requirements along with privacy regulations [9]. Different types of patient data that exist between institutions create problems with model applicability. The excessive imbalance between IDC patient classes within datasets creates problems of overfitting which makes a model succeed in existing data yet fail with new cases. Deep learning systems currently have poor interpretability which causes practitioners to be reluctant to utilize them in their practice. The opaque decision-making system of CNNs makes it hard for regulators to grant approval for healthcare applications because transparency remains essential for medical decisions. Consumers equipment along with substantial computational resources that clinical facilities typically lack leading to additional deployment period [37].

The integration of DL for IDC management proves challenging when WSI is not available as a processing method. The collection of data faces regulatory barriers because of privacy laws which negatively affects the amount and standard of annotation data. The differences between medical images during training create challenges for model performance since they hinder generalization abilities. The absence of explainability in diagnostics remains a problem for clinicians in critical healthcare scenarios although DL systems operate without transparency [38]. A major limitation in IDC detection through deep learning involves execution complexity which leads to delays in clinical applications because of latency limitations and demands powerful GPUs for training. Systemic ethical issues together with legal challenges emerge because deep learning models absorb preexisting biases from their training sources leading to unfair outcomes. Security protection for data together with proper regulatory compliance constitutes an essential necessity. The substantial development of neural networks requires joint efforts between pathologists and data scientists and regulatory experts for building resilient and interpretable generalizable systems [39].

Supervised learning remains the standard approach for conducting analysis on WSI within IDC detection research. The detection method depends on extensive labeled data during training for devising models that identify malignant cells from benign ones. Foundational CNN architectures continue being effective for medical imaging diagnosis purposes as they help pathologists in their clinical decision-making processes. Multiple Instance Learning (MIL) helps WSI analysis by automatically detecting cancer regions despite having no requirement for pixel-level markings [40]. The diagnostic accuracy gets improved by MIL while also reducing the complexity of tasks for pathologists. Pre-trained models bring benefits to pathology work. The initial training of models on various types of samples makes possible their subsequent modification for targeted applications. The improved data efficiency together with shorter development periods are the advantages that result from this approach. The deployment of real systems needs to address three critical factors: domain adaptability, interpretability and ethical considerations. Computerized pathology analysis requires enormous quantities of well-documented datasets alongside its demanding computational requirements [41]. Through self-supervised learning models obtain preliminary training that minimizes the effects of overfitting during classification using few instances of data. Computationally expensive is the cost of running Model-Agnostic Meta-Learning (MAML) and similar meta-learning techniques. The effectiveness of Vision Transformers remains steady despite their sensitivity to overfitting problems with limited available data. The implementation of clinical integration requires extensive testing of data sources which differ from each other [42].

The classification of WSI improves significantly with models built upon Transformer architecture because they understand spatial patterns within WSIs along with temporal connections [43]. They need substantial resources for instructional and prognostic processes. The continued presence of data bias along with the lack of interpretability leads to problems when attempting clinical deployment. The development of AI pathology depends on resolving these identified problems [44].

The proposed **global contrast-masked autoencoder (GCMAE)** combines masked autoencoding (MAE)—which reconstructs masked histopathology patches to learn local features (e.g., cellular patterns)—with global contrastive learning to capture holistic semantic distinctions (e.g., tumor vs. normal tissue). This dual approach enables hierarchical representation learning, integrating fine-grained reconstruction with tissue-level context. Evaluated on TCGA and Camelyon16, GCMAE outperforms self-supervised (MoCo, SimCLR) and supervised baselines in cancer subtyping, tumor detection, and survival prediction, achieving state-of-the-art accuracy with minimal labeled data. The framework highlights the value of merging reconstruction and contrastive objectives in medical imaging, offering a scalable, annotation-efficient solution for computational histopathology [45]. This work introduces **pseudo-data based self-supervised federated learning (PDS-FL)** to address data privacy and annotation scarcity in histopathological image classification. The framework leverages **synthetic pseudo-data**, generated to mimic real histopathology features, to pre-train models across decentralized medical institutions via federated learning (FL). A self-supervised task (e.g., contrastive learning or puzzle-solving) is applied to both pseudo-data and unlabeled client data, enabling robust feature extraction without compromising patient privacy. By mitigating data heterogeneity and label dependence, PDS-FL achieves competitive accuracy in tumor grading and subtype classification on benchmarks like TCGA, outperforming conventional FL methods (e.g., FedAvg) and centralized self-supervised baselines. The approach demonstrates scalable, privacy-preserving collaboration for medical AI, reducing reliance on annotated datasets while maintaining diagnostic precision [46].The Table 1 provide the summary of these related work with approaches, dataset, key findings, advantage and limitation/future works.

**Table 1:** Summary of related work for image disease Identification and classification.

Reference	Model/Approach	Dataset Used	Key Findings	Advantage	Limitations/Future Work
[24]	**AAU-Net with HAAM** (Hybrid Adaptive Attention Module)	Three publicly available breast ultrasound datasets	Achieved superior segmentation accuracy for intricate breast lesions	Improved feature extraction through HAAM, better representation of complex lesions	Needs further validation on diverse real-world datasets
[25]	**SDFNet (Structural & Detailed Feature Fusion Network)**	Renal ultrasound dataset	Outperformed other methods in kidney segmentation	Enhanced boundary detection via BD module	Requires optimization for higher-resolution ultrasound images
[26]	**Iterative Up-Sampling Optimization Strategy**	KUS dataset	Jaccard: 89.95%, Dice: 94.59%, Accuracy: 94.47%, Recall: 95.07%, AUC: 0.9703	Reduced model complexity while maintaining high segmentation accuracy	Performance may vary with different medical imaging datasets
[27,28]	**Refinement Residual Convolutional Network** (SegNet with deep supervision, missed & false detection residual networks)	Two public breast ultrasound datasets	Demonstrated superior segmentation accuracy compared to existing methods	Effectively reduces false/missed detections	Requires higher computational resources
[29]	**Cascaded CNN (U-Net, BAGNet, RFNet)**	BUSIS dataset	Outperformed other methods in lesion segmentation	Hybrid approach enhances lesion detection accuracy	Needs optimization for real-time applications
[30]	**Telecare-Based Telemammography (CEDM)**	Rural Indian women dataset	Improved early breast cancer detection through telemammography	Enables remote cancer screening for underserved populations	Requires further integration with AI-based decision support systems
[31]	**CNN Deep Feature Fusion + ELM Clustering**	Breast cancer dataset	Achieved efficient and accurate mass detection and classification	Enhances feature representation by combining clustering and deep learning	May need additional clinical validation on larger datasets
[32]	**Comparison of ANN, MLP, and CNN**	Standard test dataset	Evaluated performance of different models for early-stage breast cancer detection	Comparative study highlights strengths and weaknesses of each model	Needs further study on feature interpretability and explainability
[33]	**Transfer Learning-Based Deep Learning (GoogleNet, ResNet, VGGNet)**	Breast cytology images	Outperformed previous methods in detecting malignancy	Transfer learning enhances classification accuracy	Requires larger dataset for improved generalization
[34]	**Ensemble Deep Learning (VGG-16, VGG-19)**	Histopathology images	Achieved high accuracy (95.29%) and sensitivity (97.73%) for carcinoma classification	Ensemble models improve classification robustness	Computationally expensive; needs optimization for real-time diagnosis
[36]	**Double-Shot Transfer Learning (DSTL) with Data Augmentation**	X-ray breast cancer dataset	Enhanced precision and efficiency of breast cancer categorisation using pre-trained models	Prevents overfitting, enhances model adaptability using domain-specific fine-tuning	Requires further validation on diverse datasets and real-world clinical settings
[37]	**Transfer Learning + Deep Learning Model for Breast Cancer Diagnosis**	Breast cancer dataset (80–20 cross-validation)	Developed customized deep learning architectures for automated cancer detection	Enhances generalization using cross-validation and problem-specific architectures	Needs optimization for real-time applications and handling high-resolution images
[9]	**Deep Learning (DL) for IDC Detection**	Medical image datasets	Identifies challenges in data complexity, dimensionality, and overfitting due to dataset bias	Effective in IDC identification and classification	Requires high computational power, limited interpretability, and difficulty in clinical adoption
[38]	**Deep Learning for IDC Detection Beyond Whole Slide Imaging (WSI)**	Annotated histopathological images	Highlights the importance of high-quality, diverse medical datasets for robust DL models	Improved model robustness across demographics	Model bias due to population-specific training, difficulty in real-world generalization
[39]	**Deep Neural Networks in IDC Classification**	Histopathological images	Discusses ethical and legal concerns in IDC detection using DL	Enhances diagnostic precision	Requires improvements in transparency and regulatory compliance
[40]	**Multiple Instance Learning (MIL) for IDC in WSI**	Whole Slide Imaging (WSI) dataset	Uses MIL to detect cancerous regions in histological images	Helps in detecting malignant behaviors in IDC	Lacks pixel-level annotation, requiring further model refinement
[41]	**Pre-trained Backbone Models for Computational Pathology**	Digital pathology datasets	Highlights the benefits of pre-trained models in IDC detection	Reduces training time and enhances model adaptability	Requires domain-specific adaptation and ethical considerations
[42]	**Million-Slide Digital Pathology Foundation Model**	Large-scale digital pathology dataset	Enhances IDC classification accuracy using pre-trained models	Versatile across different settings and pathology applications	Requires high-quality annotated data for effective model performance
[43]	**UNI-based AI Pathology Solutions**	Computational pathology datasets	Develops a memory-efficient and adaptable architecture for IDC detection	Computational efficiency and model adaptability	Computationally intensive, requiring significant infrastructure and resources
[44]	**Dual-Channel Prototype Network (DCPN) with Pyramid Vision Transformers (PVT) & CNNs**	Pathological image datasets	Enhances model generalization and improves disease classification in limited data settings	Learns from minimal data, multi-scale feature extraction, high resistance	High computational cost, requires high-quality annotated data, interpretability challenges
[38]	**Deep Reinforcement Learning (DRL) for Melanoma Detection**	Whole Slide Imaging (WSI) datasets	DRL enhances melanoma detection efficiency, aiding pathologists in workload reduction	Rapid processing of large WSI datasets, improved diagnostic accuracy	Computationally expensive, sensitive to data quality, risk of overfitting with small datasets
[47]	**Transformer-Based Models for WSI Classification (Multi-Instance Learning – MIL)**	Large-scale WSI datasets	Transformers improve spatial-temporal correlation learning for medical image analysis	High classification accuracy, robust feature extraction	Computationally intensive, resource-demanding, complex clinical implementation

## 3. Problem statement and research motivation

### 3.1. Research contributions

By utilizing the methods or techniques, these research gaps can be filled, or identified limitations can be overcome. The following is the main takeaway from this research:

- Breast cancer histopathology picture categorization benefits from the discovery of relevant IDC datasets.
- To learn more about methods for improving medical pictures via preprocessing before using them in histopathology scans.
- To close the research gaps and examine and assess how histopathological image data processing affects the detection of breast cancer.
- Offer a customized CNN model for training and determining the breast cancer categorization.
- To calculate the performance outcomes of the proposed model on the IDC dataset using performance measures according to “acc-uracy, pre-cision, re-call, and f1score”.
- After obtaining the proposed results, existing models are compared to the proposed model.

## 4. Research methodology

To solve the challenging issues in breast cancer classification, especially data imbalance problems, is a big concern nowadays. The first phase of this work is collecting data on breast cancer classification. This analysis utilizes the freely accessible IDC dataset, which contains unbalanced data. Next, resize the image into 32x32 pixels in the data preprocessing. As discussed above, the collected dataset is unbalanced. A SMOTE class imbalance approach has been suggested to address this problem. CNN was then established as a special class of models for challenges involving object recognition. To assess images, CNN, a deep learning model, extracts visual features. To perform the classification, a custom CNN model is built with a different number of layers. Another classification approach requires eliminating an illustrative feature using a feature extraction technique. In this study, 2 categories must be classified as IDC positive and IDC negative.

The study’s main objective is to categorize images into two groups: IDC positive and IDC negative. IDC, which is a prevalent form of breast cancer. The objective is to create a system capable of precisely differentiating between photos displaying evidence of IDC (IDC positive) and those that do not (IDC negative). Image Segmentation: Prior to neural network training, the research isolates image segments. This probably requires pre-processing to separate certain areas or characteristics in the pictures that suggest the presence of Invasive Ductal Carcinoma (IDC). The segments are then used as input for analysis. Evaluation of CNNs performance in a classification challenge. CNNs are often used in image processing applications because of their capacity to record spatial hierarchies of data. The article implies that CNN’s performance in this research surpasses the results achieved by other picture classification methods on various datasets.

**Fig 1:**
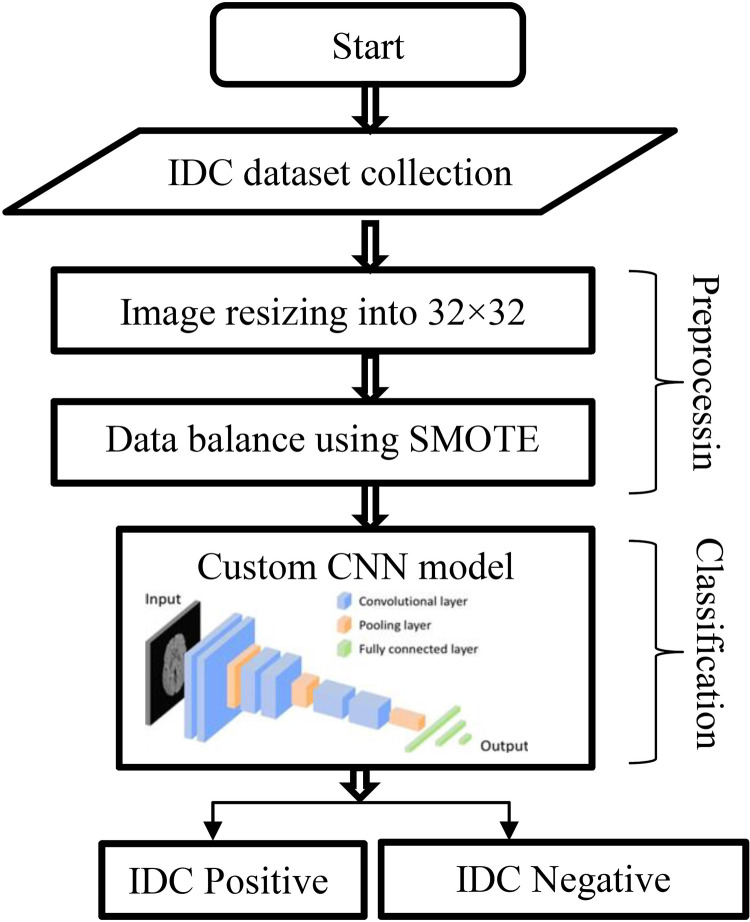
Flowchart of Proposed SMO_CNN.

**Technique Overview:** The suggested technique is presented in a flowchart, likely seen in Fig 1. This flowchart outlines the procedure into four steps, which will be elaborated on later. Overall, and presented in the Fig 1 outlines the objectives, methodology, and for each step separately to provide a thorough knowledge of the methods used in the research. To classify breast cancer images as IDC positive or negative, we provide a synopsis of the study’s objectives, methodology, and comparative outcomes in developing a CNN-based system. It also demonstrates a systematic method of thoroughly describing the process by outlining the several steps.

Class imbalance does not only mean there are more instances of one class than the other but it goes a step further to imply that the ratio of the two classes is not ideal. IDC identification Inherently Complex: While it is accurate that this database may contain over 70k images of IDC (Invasive Ductal Carcinoma), it is for this reason that the IDC’s are not solely determined by quantity. The presented approach uses LPBC to synchronize picture hallucination with difficult IDC identification as it does not contain normal tissue and has small morphological differences between malignant and benign tissues. The contrast-enhanced picture assists the diagnosis; nevertheless, even these images do not match the variety that distinguishes normal tissue from the tumor. Another key aspect that should be tended to is to prevent the generation of an over-complex and fragile model if the dataset is significantly large. This means has to check that the model has not deteriorated and that it feasible to predict new unseen data points. However, depending on the actual setting of a clinical context, the imbalanced-data challenges might encompass a variety of aspects and can result in either overfitting to the majority’s class or generalization.

### 4.1. Significance of sophisticated strategies

SMOTE-based Performance Enhancement: Some of the measures that can be taken in the future include the usage of method like SMOTE that seeks to balance for the classes that have a small number of instances to increase the chances of the model performing better in future. This is to get a training set that has a more equal distribution of objects to the classes hence better training of the model. Moreover, it enables such uncommon subtypes of IDC that are not seen often and may not be included in the data set.

Therefore, the application of such sophisticated models as transformers or pre-trained models, as well as self-supervised learning ensures that such models are not only general but tuned to identify intricate patterns concerning histopathology pictures. This brings about more precision and reliability of the model’s diagnosis. Although it may seem that the number of samples is quite adequate for training a deep learning model, much more critical are the facts that the classes are distributed extremely unequally (their rate is less than 2%); it is necessary to take into account geographic environment; there are seen clusters in AUC ROC curves; there are challenges related to the clinical practice. It allows for the creation of models that are accurate, reliable, and feasible in a wide range of actual clinical settings, which ultimately improves patients’ long-term well-being.

### 4.2. Data collection

Collecting data allows a person or group to assess results, forecast trends and probability, and answer critical questions. Accurate data acquisition is crucial for preserving research integrity, making better business decisions, and ensuring quality control.

**Fig 2:**
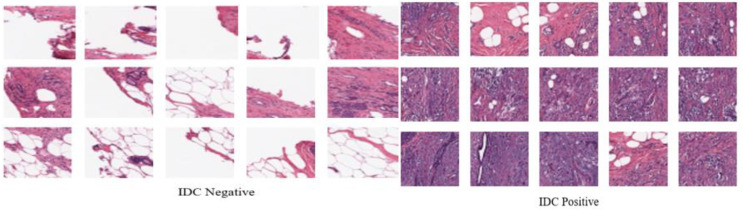
Sampling Images of IDC Dataset.

This work utilized the histopathology imaging dataset, the IDC dataset, to perform this task. It is a well-known collection, including IDC positive and negative images that are freely accessible to the public. The IDC dataset contains 277,524 color photographs, where 1,98,738 (71.61%) of these images are classed as IDC (-) and 78,786 (28.39%) as IDC (+). However, this dataset is substantially unbalanced. The sample pictures for the IDC dataset are represented in Fig 2.

### 4.3. Data preprocessing

When passing images to a higher level of abstraction, pre-processing is performed at the lowest degree of error feasible, with both the input and output images becoming intensity images. The iconic images are astonishingly similar to the unprocessed sensor data. The intensity image is a matrix of image function values, not an image. The fundamental goal of pre-processing is to raise the quality of the picture collection by removing unnecessary changes and improving some visually appealing traits [47]. Image preparation, before they are used in modeling training and validation is known as image pre-processing (IP). This process covers resizing, orientation, and color adjustments. On both the training and test sets, pre-processing image processes are applied. The pre-processing step is required to prepare image models from data inputs. For example, fully connected layers in CNNs require that almost all images be equal arrays in size. In addition, pre-processing images may speed up model conclusions and shorten training time. In cases when the input photos are very big, decreasing their size significantly lengthens the training period of the model without negatively impacting its performance. Pre-processing procedures are performed at the lowest possible error level when images are translated to a higher level of abstraction and the both the input picture and the output picture are converted into intensity images. As for the two sets of raw sensor data and the iconic photos, they look rather alike. In this case, image function values are matrixes creating what is known as the intensity image and it is not a picture. In summary, the goals of pre-processing are to reduce the amount of inevitable alterations and enhance particular, beneficial for visualization, characteristics, while collecting a set of pictures [48]. Preprocessing or IP is the general procedure through which images are prepared before they are employed in model training and testing. Several of these methodologies include but not limited to resizing, flipping and changing color. Before analysis, images go through pre-processing to the training, and the test datasets. Indeed the pre-processing stage is very important and is needed in order to be able to make picture models from raw data. For instance, nearly all the photos ought to have the same array size for CNNs’ fully linked layers to be functional. Also, it is possible that pre-processing images may help to reduce model’s conclusions and training time. The above update greatly enhances the model’s training time if the input photos are quite massive without a change in performance when the images are shrunk to a smaller size.

Nevertheless, one will have to admit that there are actually quite a few things one should take into consideration when scaling images. Square input photos are needed by many model designs; however, there are only a few technologies that can properly capture square pictures. If one wants the picture to be completely square, then one. While resizing images may seem straightforward, many factors need to be considered. Numerous model designs need square input images, but only a few technologies record square images correctly. In converting an image to a squared form, the dimensions must be expanded to suit, or its aspect ratio must be maintained. At the same time, additional pixels are added to fill in newly produced “dead space.” Furthermore, input images could be of varying sizes, some lower than the required input dimension.

1) **IMAGE RESIZING**

Resizing an image is a challenging task for many people. Although most images aren’t the exact size we require, it’s critical to grasp how to adjust an image effectively. Whenever an image is scaled, the pixels are adjusted. Image scaling is required when the overall pixel value has to be increased or decreased. Image resizing is the process of scaling images. It assists in lowering the number of pixels in a picture, which offers various benefits. It may shorten the training time of a CNN because more pixels in an image correspond to more input nodes, thus increasing the model’s complexity.

Additionally, this process helps in image zooming. The picture should be resized either by shrinking or scaling it up to match the size constraints, and OpenCV offers an interpolation technique for resizing an image. In this case, the image was resized into 32 × 32 pixels, as previously described [48]. Moreover, the given breast pathology images are huge, totaling 2,048 × 1,536 pixels, then selected patches out of each image and scaled them into the selected pixels extracted patches to overcome the issues of large image dimensions and inadequate data. This method was chosen to solve the large memory size in processing it. The image size is too small, and that causes much information to disappear. The only reason to select this image size is only the memory issue. There was no sufficient memory for processing a large image size, so it has been processed. If we had taken a 2,048 × 1,536 image size, it would have required more resources and time to run. And we lacked resources, so we only took a 32 × 32 image size to execute our research. Resizing is something where many people have issues on how to go about it. Strangely enough, most of images s you will be working will not be the right size so you always will need to crop and resize. Pixels are shifted every time when the picture is altered. For augmenting or diminishing the overall value of the pixels, image scaling is required wholly. Resizing photos simply means establishing a new dimension of pictures. It assists in decreasing the pixel density that is implemented in an image and this has the following benefits. The training time of a CNN may be cut short if this would happen as the model becomes more complex as the input nodes in an image increase.

Synchronization with the video or other images also enables the zooming of the figures to be enhanced. This has to be fit into the size restrictions, and that means that the picture has to be either scaled up or down. OpenCV has one interpolation technique to address this issue. The picture was blurred in the same manner as before and reduced to 32 × 32 pixels in this case. Moreover, the scale of the pictures of breast pathology is very large and they have dimensions of 2048 × 1536. Concerning with the issues stemmed from high size of photos and lack of data, each photograph was reduced into patches that correspond to the given pixels. This approach was chosen because to perform it, one needs a virtually unlimited amount of memory to work with. Because the image is small only a limited number of details can be made out. This picture size was opted for only in consideration of the memory usage and no other reasons. It has been processed since there was a problem of inadequate RAM to support a large picture size. The time and resource usage would have been higher in case we used the image size of 2,048 × 1,536. Thus, because of lack of enough resources we were compelled to work with only a 32 × 32 picture size for our study.

2) **DATA BALANCING: SMOTE**

In real-world issues, an imbalanced dataset is not scarce. The essay introduces the notion of an unbalanced dataset, a prevalent occurrence in real-world situations. An unbalanced dataset is characterized by an unequal distribution of occurrences across different classes. Put simply, some classes may have a notably larger number of samples than others. Imbalance in data may create difficulties in classification tasks since the model could exhibit bias towards the majority class, resulting in subpar performance in predicting the minority class. We have used the SMOTE technique to tackle unbalanced data in the context of 2D picture data. SMOTE is a method that creates artificial samples for the underrepresented class by filling in the gaps between current samples. By balancing the distribution of classes in the dataset, it enhances the model’s capacity to learn from the minority class. The 2D data is image converted to 1D data prior to using the SMOTE method. This procedure probably includes converting the picture arrays into one-dimensional vectors, which is a typical preprocessing step in ML projects that deal with image data. The SMOTE is used on an imbalanced dataset after converting the data to a one-dimensional format. Artificial data points are created for the underrepresented class, hence boosting its presence in the dataset. After using SMOTE and balancing the dataset, the 1D data is transformed back to 2D data. This phase is essential to reestablish the initial format of the picture data, guaranteeing it aligns with the neural network architecture created for analyzing 2D images. We used the SMOTE technique to tackle unbalanced data in 2D image classification, enhancing the robustness and accuracy of model training.

Before digging into the processing of unbalanced data, the problems that an imbalanced dataset might cause should be understood. When dealing with real-world issues, an unbalanced dataset is highly prevalent. A ML model is not resilient if trained on an imbalanced dataset. Therefore, Machine learning models should be trained on a balanced dataset. Balanced data is created using methods such as SMOTE.

The SMOTE algorithm begins before dividing the data since the data utilized wasn’t balanced. Hence, the oversampling method of the SMOTE algorithm was employed to obtain the data balance. After partitioning the data, a lengthy process would have to be followed to perform the SMOTE algorithm. The data would be partitioned into three categories: training, testing, and validation. In this case, the SMOTE technique is applied individually three times on these three data, so performed the SMOTE algorithm before splitting the data. This approach may generate as many synthetic examples as needed for the data. The research suggests using random under-sampling to lower the majority class’s occurrences, and then SMOTE to increase the minority class’s occurrences, in order to achieve distributional parity. The combination of SMOTE with under-sampling outperforms simple under-sampling. To address this, we will revise our methodology as follows: To this, we shall change the following in the new methodology:

- **Training Set:** As it was stated, handling the class imbalance is a sensitive matter and therefore, SMOTE technique will be used only on the training set. This is preferred as it will cause the weights to be adjusted to the features of the data nearly equally for the model to learn from.
- **Validation Set:** Regarding the former, we shall also have in place the basic data or the original data in the validation measures. This is relevant with regard to that situation where the figure of merit of the model is determined from raw data, hence showing a proof of the generalization of the model.
- **Testing Set:** Likewise, the testing set will have new untouchable data-like the training set similar to it. This will assist in laying the groundwork on the actualization of the model along with the extent of accuracy that it would have.

Thus, it can be concluded that carrying out these two things enables adequate conditioning and construction for the model, after which it can be accurately assessed.

To oversample is to increase the level of a minority class so that is levels with that of all the values of a majority class. Thus, specific synthetic data points might be created after increase the quantity of the information in the data. One of them is SMOTE. Thus, as the name implies, SMOTE is an oversampling technique [28]. Concerning the process of synthesizing the data, the SMOTE technique includes a k-nearest neighbor approach. The first approach adopted by SMOTE was expressed as the random selection of data, which was chosen from the minor class. The KNN of the data is then obtained using the function as the name suggests. Having these, this sample size would also be combined with equally randomly selected, to construct the synthetic data. Then, SMOTE was utilized to train a dataset using the Imblearn library.

Thus, the use of SMOTE method in the computational pathology model for training enhances the diagnostic accuracy, model generalization, patient care results, efficient use of clinical resources and enhancement of research and development. It is indisputable that these advantages enhance patient’s care and the overall effectiveness of health sectors. In machine learning datasets, this technique handles class imbalance it is called smote Computational Pathology Applications: SMOTE algorithm is utilized for computational pathology whereby has the following clinical implications Increased Diagnostic Accuracy: Balanced Training Data: It synthesizes examples of the minority set by creating new synthetic samples for the minority class, thus helping to balance our training data, another step up from the prior model. This increases the efficiency in model learning and decreases prejudice against minorities. New algorithms in the image classification may perhaps augment the diagnostic accuracy in clinical application by diagnosing less frequently encountered disease or different types of cancer. Perhaps due to their small sample size, it is even more crucial to reduce overfitting if the sample is compiled from minorities only. This enhances the model’s generalization of unseen data. From this, it can be inferred that the diagnostic paradigm will apply to all the other rare illness patients within clinical practice.

Oversampling is quantizing minority class values to match the total count of majority values. Synthetic data points can be created to improve the amount of information in the data. SMOTE is one of these techniques. SMOTE is an oversampling method, as its name suggests [49]. SMOTE uses a k-nearest neighbor algorithm to generate synthetic data. The initial choice made by SMOTE was random data from the minor class. The function then determines the data’s k, nearby neighbors. The synthetic data would be constructed by combining this sample size with randomly picked data from the KNN. Then, SMOTE was utilized to train a dataset using the Imblearn library. Ensuring Data Balance: Which smote me like a heavy blow on the heart Smote. The problem with skewed datasets is that there is no scarcity of such problems in real-life situations. The actual-world scenario of having an imbalanced dataset is brought into focus in this article. It is quite apparent that an imbalance dataset means that occurrences are diverse and not equal in various classifications. In other words, the extraordinary number of samples from the given classes could be much different from the number of samples from others. Because of imbalance in classes, classification tasks can get even more complex because, the model tends to favor the most common class, while performing poorly with the rare class. The method we have used to counter imbalance within the framework of 2D image data is SMOTE. SMOTE is a technique where all the empty spaces are filled with new one’s hence developing fake samples for the underrepresented class. Class balancing in the dataset means that the ratio of classes is more equal and this increases the model’s capability of learning the classes that are scarce. Before applying SMOTE, the 2D data is image converted to 1D data for preparing it towards the technique. Given that this can be considered as a standard preprocessing step in many ML projects where input data is represented as images, this process is most probably connected with converting multi-dimensional arrays where images are stored into one-dimensional arrays. Once the data has been converted to one dimensional representation, it is used for applying SMOTE on an unbalanced class problem. Since the aim is to include all aspects of the underrepresented group, artificial data points are added to its profile in the dataset. Indeed, after SMOTE, we transform the 1D data back to a 2D data set. Since the identified architecture will be used to analyze 2D images, it is necessary to ensure that it is suitable for the restored original picture data format. To enhance the quality of training model for cutter, we applied the SMOTE method to solve the problem of imbalanced data for 2D image categorization. Especially, one must be aware of the pitfalls that may occur due to an imbalance dataset before moving on to the processing of such dataset. When solving real world problems, it is often a norm to find a set which has been skewed in its distribution. Due to this, if a machine learning model is trained on an uneven data set – intentionally, it will not be robust. Hence, a balanced dataset is preferable to be used in building machine learning algorithms. Such methods include, SMOTE that is utilized in the generation of balanced data. As the data we used was not balanced, the SMOTE is commenced before the data splits. Therefore, for obtaining the data balance, the SMOTE algorithm’s oversampling technique was applied. A time-consuming procedure would be required to be done to apply the SMOTE algorithm after the data division. The overall set of data would be divided into a training set, a test set and a validation set. Thus, before splitting the data, the SMOTE algorithm was applied to each of the three sets of input data three times. Thus, there is the possibility of creating an almost infinite number of synthetic cases based on data-driven ones. To reduce the occurrences of the majority class, the research recommends the use of random under-sampling, and to increase the occurrences of the minority class, SMOTE should be applied to level the dataset’s distribution. The studies have indicated that by under-sampling and using SMOTE, an improvement is achieved as compared to under-sampling alone would need to make it bigger technically or simply maintain the aspect ratio. At the same time, more pixels are encouraged to cover the newly appeared ‘blank zone’. Besides, the input images may not be of the same size as some of them may contain a smaller resolution than the required input size.

**How to balance the dataset:**

The SMOTE algorithm’s processes are as follows:

1 Determine the vectors for minority classes.
2 Determine the number of (k) nearest numbers to consider.
3 Incorporate a fusion data point by linking minority data points to any neighbouring data point.
4 Recall these steps for every minority data point and all k neighbours until the data is not balanced.

3) **CONVERSION: CONVERSION INTO TENSOR (ARRAY) FORMAT**

A function named “convert_to_tensor” in Tensorflow is used to carry out this process, and it converts the input value into an appropriate tensor. The value may be a NumPy array, a Python list, or a set of Python scalars; however, for this section, the function will return a tensor. Alternately, a function can convert an array into a tensor several times.

This Table 2 summarizes the key data preprocessing steps applied to the IDC histopathology imaging dataset before training the deep learning model. The preprocessing techniques include image resizing, data balancing using SMOTE, and conversion into tensor format. These steps ensure uniformity in image dimensions, mitigate class imbalance, and prepare data for model training and validation.

**Table 2:** Data Preprocessing Steps for IDC Dataset.

Step	Description	Purpose	Techniques Used
**Dataset Overview**	IDC dataset (277,524 images) with 71.61% IDC (-) and 28.39% IDC (+)	Highlight data imbalance	IDC dataset (open-source)
**Image Resizing**	Resizing images to 32 × 32 pixels from original 2048 × 1536 pixels	Reduce computation cost, ensure consistency	OpenCV interpolation
**Data Balancing (SMOTE)**	Addressing class imbalance by generating synthetic minority samples	Prevent model bias, improve classification performance	SMOTE
**Conversion to Tensor**	Transforming image data into tensor format for deep learning models	Ensure compatibility with TensorFlow	convert_to_tensor function in TensorFlow

### 4.4. Dataset split

**Fig 3:**
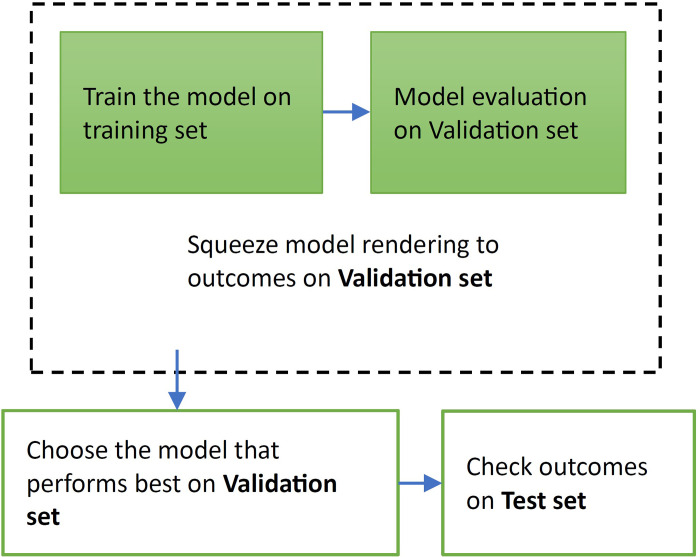
Training Data/Validation/Test.

A dataset’s split ratio is determined by the amount of sample data and the model (as shown in Fig 3). The data was separated into three distinct dataset splits for training and testing our model, as shown below:

1. Training (60%): No. of images: 45,945
2. Validation (20%): No. of images: 14,358
3. Testing (20%): No. of images: 11,487

The word “training data set” refers to samples utilized to perform training on the model. The samples used to assess performance are referred to as “test” or “validation data set” in contrast. The dataset that has traditionally been used to evaluate the final model’s performance is known as a “test set.” Training data is composed of data on whose practical training is performed. The validation set might be regarded as a subset of the training data set because it is utilized to implement models in this project, such as CNN. Fine-tuned models may improve model performance after each epoch with validation splits. The testing dataset tells us more about the model’s final accuracy when the training/learning phase is completed [50].

**Training Set:** Latent attributes in the dataset are trained and discovered using the data set. The CNN is fed data that is very similar to the previous training dataset at the beginning of each new epoch, and also, the models continue learning the data features. The training set requires various inputs to guarantee that the classifier is trained in all potential scenarios and can detect any previously unrecognized sample data. When the input images are exceedingly huge, lowering their size significantly boosts model training time without compromising performance.

**Validation Set:** The validation set is different from the training dataset. In addition, it is used to evaluate the efficacy of the proposed scheme as it is being trained. This validation strategy generates data that is used in fine-tuning the model’s hyper-parameters and settings to follow the particular requirements. This process is analogous to obtaining feedback about whether our training is on track. The model was trained on one data set and then tested on another set of data, called the validation set for each iteration. To avoid model overfitting, the dataset was split into a training set and a validation set, which occurs whenever the model has become excellent at categorizing the sample data in the training set and cannot generalize and produce realistic categorizations on data that has not been seen before.

**Test Set:** Once the model has finished the training phase, it must be evaluated using a distinctive data collection. It results in an unambiguous final model performance measure of precision and accuracy.

### 4.5. Training model: Customized CNN

A customized CNN model is used to perform training and obtain the classification of breast cancer. Among other tasks, IDC classification is made possible with the use of CNNs, which stresses the utilization of original input data with minimum feature engineering. Pattern-specific feature maps are utilized to construct feature maps; lower layers in the network recognize properties similar to those in input images, whereas deep levels detect abstract shapes.

**Fig 4:**
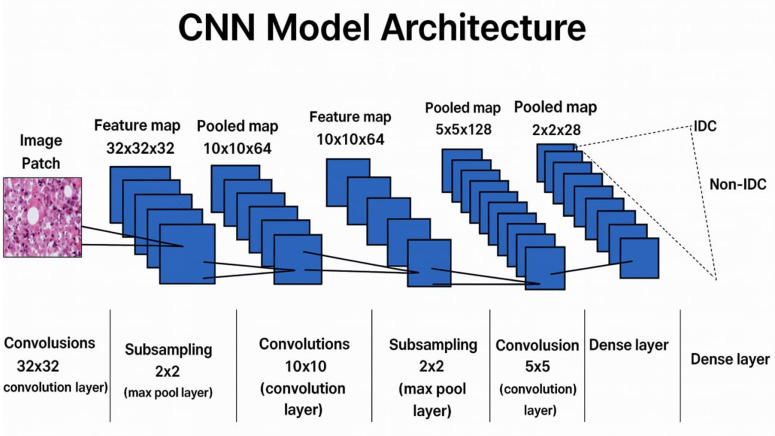
CNN Model Architecture.

Feature maps could be flattened and divided into dense layers, which can be compared for classification tasks. CNN is the most popular deep learning architecture to solve an image classification problem (Fig 4). This research aims to identify the category to which the input image pertains. A CNN is constructed in four basic phases as follows [51]:

1. Convolution.
2. Pooling.
3. Flattening.
4. Dense (Fully Connected) layer.

The reasons for splitting our data into training, validation, and test sets are outlined in Table 3. The ultimate goal of any machine learning model is to generalize its acquired knowledge to instances it has not yet encountered. Fundamentally, we should train on a subset of our entire dataset, reserving the remaining data for assessing the generalizability of the model.

**Table 3:** Hyperparameter Tuning of the Proposed Model.

Hyperparameters	Number of Counts
Convolution Layer	5
Max Pooling Layer	3
Batch Size	128
Name of Optimizer	3 (SGD, Adagrad, & Adam)
Epochs	50

1) **Types of Layers**

All neurons in a layer perform mathematical operations identical to one another, through which the layer gets its name.

**Convolution layer:** Convolution is the computational method used to filter inputs and recognize patterns in information in image processing. In this layer, convolution is applied to all the neurons’ inputs. The filter size of a convolutional neuron is the most critical metric to consider. This output will be calculated over the whole image by sliding the convolution filter across it and the window slide set by one pixel at a time, and this number is mentioned as the Stride value. Typically, more than one filter is employed in a single convolution layer, a common practice.

**Pooling layer:** It is often implemented immediately after the convolutional layer to decrease the feature’s dimension of the outcome (only width and height, except depth). As a consequence, the set of parameters and computation time is reduced. Using fewer parameters also helps avoid overfitting. The most often used method is max pooling, which involves taking a filter with dimensions of 3 × 3 and performing the maximum operation over a 3 × 3 sized image section.

**Dense (Fully-Connected) Layer:** Every neurone in a “fully connected layer” receives input from all neurones in the layer below it, just as in the previous layer. In this layer, the output is produced by matrix multiplication followed by a bias offset on input.

2) **Dropout**

Dropout [52] randomly prunes the model after each training period. Ablated neurons are accounted for in the model by resetting all weight values in the node at random intervals to 0, thus allowing the model to learn. This randomized ablation reduces model overfitting, allowing models to transfer findings to validation and test data more precisely. This procedure results in a more stable CAD model, which enhances the performance of domain-specific and maybe cross-domain data [53].

3) **Batch Normalization**

Batch normalization [54] minimizes the dependency of the activation function upon scaling parameters or starting values that promote self-regularization. This process regularizes extraordinarily large values into a narrower context [55,56]. Batch-normalized models operate at a faster learning rate because each layer rapidly acquires a more stable parameter distribution.

4) **Optimizer**

Optimizers are used to numerically reduce model loss functions by back-propagation, a technique known as optimization. Back-propagation adjusts the model’s weights in response to errors compared to ground-truth images (loss). Numerous optimization techniques have been utilized, including Stochastic Gradient Descent (SGD) [57], AdaGrad [58], and Adam [59].

Research scholars [56–61] continue to employ the traditional SGD, while dynamic optimizers such as Adam are getting popular. Thus, the Adam optimizer is employed because it dynamically adjusts the learning rate using gradient momentum.

Adam is an extension of the SGD optimization approach, which has lately received widespread use in DL applications, including computer vision and NLP. Adam is an alternative to the standard SGD algorithm. Throughout the training phase of stochastic gradient descent, all weight updates are performed at a constant alpha learning rate.

Every network weight (parameter) has a learning rate maintained and altered independently as learning occurs. The method estimates the first and second moments of gradients and uses those estimates to calculate customized adaptive learning rates for each parameter value [62,63]. The Adam can be seen as a combination of the benefits of two different SGD enhancements. Particularly:

**Adaptive Gradient Algorithm (AdaGrad)** can effectively keep a constant learning rate for each parameter while improving efficiency on issues with sparse gradient through the AdaGrad algorithm (e.g., NLP and computer vision problems).

**RMSProp (Root Mean Square Propagation)** is a method for learning parameters that also retains learning rates for each parameter, updated depending on the average magnitude of new gradients for weight parameters (for example, how rapidly it varies). The method outperforms the competition on both online & non-stationary issues, which are a positive indicator (for example, noise).

Adam understands the advantages of both RMSProp and AdaGrad.

While RMSProp adjusts learning rates of the parameters depending upon the average 1st moment (mean), Adam additionally considers the average of 2nd moments of gradients, similar to RMSProp’s approach (uncentered variance).

Consequently, the gradient and the squared gradient are combined to create an EMA (exponential moving average). Both moving averages’ decay rates are controlled by two parameters, beta 1 and beta 2. Since the initial value of MAs and the 1 and 2 values are close to 1.0 (as suggested), the moment estimates are skewed toward zero. This bias is eliminated at first by computing biased estimates, followed by bias-corrected estimates [50].

5) **Loss Function**

Binary classification is a challenge in which we must categorize the data into one of two categories based on their attributes.

This study calculated the suggested deep learning model’s loss using the Binary cross-entropy loss function.

Binary cross-entropy loss is best comprehended once the loss function has been learned. The loss function measures model prediction accuracy. In cases where model predictions are nearest to real values, the loss will be the smallest; in cases where the forecasts are completely different from the original data, th loss value will be the greatest. This article will explicitly discuss binary cross-entropy, commonly named log loss; this is the most commonly used loss function in binary classification problems and is explored in depth below. Each projected probability is compared to the actual class outcome, which might be 0 or 1. Then, it computes the score, penalizing the probabilities based on how far they deviate from the projected outcome. This score represents how near or how far the estimated value is from the actual value.

An image height, width, and color channel are all tensors of form that a CNN accepts as input, regardless of the batch size. Regarding these dimensions, color channels relate to the number of colors seen as (R, G, B). CNN is configured in this model to process shape inputs (32 × 32 × 3), which corresponds to the format of histopathology images.

Considering this, it sends the input-shape parameter to the first layer; this process was accomplished. The output of every MaxPooling2D and Convl2D layer is a 3D shape tensor (width, height, shapes), as shown in the model summary in Table 4 (below).

**Table 4:** CNN Model Layers Summary.

Layers (types)	Output_Shapes	No. of Parameters
convl2d_5 (Convolution2D)	(32 × 32 × 32)	896
Activ_7(Activate)	(32 × 32 × 32)	0
bat_normaliza_6 (Batch_Normalization)	(32 × 32 × 32)	128
max_pool2d_3 (Max_Pooling)	(10 × 10 × 32)	0
drop_4 (Dropout)	(10 × 10 × 32)	0
convl2d_6 (Convolution2D)	(10 × 10 × 64)	18496
Activ_8(Activate)	(10 × 10 × 64)	0
bat_normaliza_7 (Batch_Normalization)	(10 × 10 × 64)	256
convl2d_7 (Convolution2D)	(10 × 10 × 64)	36928
Activ_9(Activate)	(10 × 10 × 64)	0
bat_normaliza_8 (Batch_Normalization)	(10 × 10 × 64)	256
max_pool2d_4 (Max_Pooling)	(5 × 5 × 64)	0
drop_5 (Dropout)	(5 × 5 × 64)	0
convl2d_8 (Convolution2D)	(5 × 5 × 128)	73856
Activ_10(Activate)	(5 × 5 × 128)	0
bat_normaliza_9 (Batch_Normalization)	(5 × 5 × 128)	512
convl2d_9 (Convolution2D)	(5 × 5 × 128)	147584
Activ_11(Activate)	(5 × 5 × 128)	0
bat_normaliza_10 (Batch_Normalization)	(5 × 5 × 128)	512
max_pool2d_5 (Max_Pooling)	(2 × 2 × 128)	0
drop_6 (Dropout)	(2 × 2 × 128)	0
flat_1 (Flatten)	(512)	0
dens_2 (Dense)	(1024)	525312
Activ_12(Activate)	(1024)	0
bat_normaliza_11 (Batch_Normalization)	(1024)	4096
drop_7 (Dropout)	(1024)	0
dens_3 (Dense)	(2)	2050
Activ_13(Activate)	(2)	0
Total parameters: 810,882		
Trainable parameters: 808,002		
Non-trainable parameters: 2,880		

The height and width measurements reduce as we go further into the network. In each Convl2D layer, the first parameter determines the number of output shapes available (for example, 32 or 64). As the breadth and height of a Convl2D layer diminish, we may typically afford to add additional output channels (both in terms of computation). To build the model, we feed the resulting output tensor from the convolutional base of size (5 × 5 × 128) into 1 or more than one Dense layer, which classifies the generated data. So, the current output is a three-dimensional tensor, and dense layers take one-dimensional vectors as input (Table 1). The 3D output is flattened (or unrolled) to 1D before being layered with one or more dense layers on top of it. We utilize a dense layer with two outputs at the end because IDC has two different output classes. Before passing through two dense layers, the output layers of the shape (2 × 2 × 128) were flattened into vectors of the type (512), as shown in the model summary in Table 2.

## 5. Results analysis

This section discusses the simulation environment to implement and execute the proposed SMO_CNN model on the IDC dataset. The simulation uses a Python programming environment with the Jupyter Notebook framework. A total of 50 epochs are set up in this work. Jupyter notebooks can visually show the step-by-step analysis process by organizing the various elements such as code, images, text, and output in the notebook [64,65].

It assists us in documenting the rational process as we go through the analytical process. This section consists of the configuration parameters set for the optimizer, dataset details, performance metrics, and obtained results to implement the SMO_CNN model. After that, a comparative discussion is provided to validate the model.

### 5.1. Adam configuration parameters

Adam optimization is an SGD approach based on an adaptive estimation from the 1^st^ and 2^nd^ order moments.

1. **Alpha.** This parameter is known as the learning rate or the size of the step. This value refers to the percentage of weights that have been updated (e.g., 0.001). Early learning can be accelerated using large numbers (e.g., 0.3). Small values (1.0E-5) remarkably inhibit learning throughout the training.
2. **beta1.** This parameter is callable, accepts no arguments, and returns the actual value used is examples of this type. This value estimates the first-moment exponential decay rate (0.9).
3. **beta2.** This parameter is a floating-point value, a constant floating-point tensor, or a callable that accepts no parameters and returns the actual value. For second-moment estimates, the exponential decay rate is used (e.g., 0.999). A value of 1.0 on issues with sparse gradients is recommended (for example, NLP and computer vision problems).
4. **Epsilon.** This extremely small integer prevents the implementation from dividing by zero (e.g., 1E-7).

### 5.2. Dataset

The study used the IDC dataset, a publicly available histopathological imaging collection, for conducting the studies. The collection is well recognized and consists of both positive and negative photos of IDC. The file can be downloaded from the following URL: https://www.kaggle.com/datasets/paultimothymooney/breast-histopathology-images. A dataset was generated by scanning the 162 whole slide images (WSI) of breast cancer specimens and assembling them into patches of size 50 × 50. The original collection consists of 162 Whole Slide Imaging (WSI) photographs, which were scanned at a resolution 40 times higher than the original. The photographs originated from various establishments, such as Penn Medicine and the CancerInstitute of NewJersey. The IDC dataset was created by extracting and merging several photo samples from each participant. The pathologists indicated the cancerous regions on the images after extracting them from the Whole slide imaging images. The images were divided into patches of non-overlapped RGB image data. An overall number of 277,524 color images are included in the dataset, among which 198,738 (71.61%) are classified as IDC (-).

In comparison, 78,786 images (28.39%) were classified as IDC (+), demonstrating that the collection is severely skewed. Fig 2 illustrates (+) and (-) images from the sample dataset. Each picture is called “PID idx5 X Y classK.png” (e.g., 10254 idx5 x1000 y352 class1.png), where PID indicates the patient ID (10254), X (x1000), and Y (y352) are the (x, y) coordinate from which patches are extracted, and K is a class, where 0 denotes negative findings, and 1 denotes positive outcomes.

The two datasets each for training and testing were created. Approximately 60% of the images were utilized for training, the remaining 20% for validation, and the remaining 20% for testing. Only two classes are available in this IDC dataset, classified into two categories. We have used the scaling range from 0 to 255 because I have been indexing from 0 to 255. If we indexed the values from 1, the scaling was 1–256.

The images from the initial training and testing are shown in further detail in Table 3. Based on Table 5, 194,266 pictures were utilized for training deep learning models; the remaining 83,258 images were used to evaluate the deep learning model’s performance.

**Table 5:** IDC Breast Cancer Dataset Descriptions.

Class	Original images	A training set of images	Testing set of images
IDC (-)	196,778	1,99,116	59,622
IDC (+)	78,786	55,150	23,636
Total	2,27,524	1,94,266	83,258

### 5.3. Performance evaluation metrics

Metrics that quantify performance can be utilized to assess the model’s performance. Unlike loss functions, metric functions are not employed during the model’s training; instead, the outcomes of assessing a metric are used to inform the model’s evaluation.

**Fig 5:**
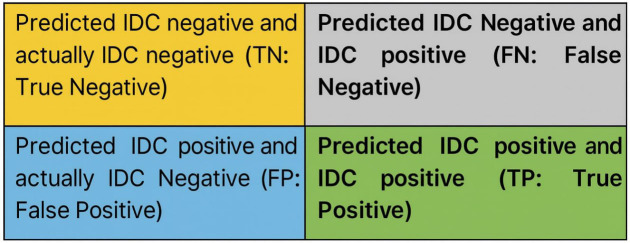
Confusion Matrix with a Description of the 4 Quadrants for Our Case.

1 **Confusion matrix:** We employed a confusion matrix to assess the model’s performance. The confusion matrix is divided into four quadrants in the binary classification matrix: TP, TN, FP, and FN. Fig 5 illustrates a simplified representation of the four quadrants of the confusion matrix in our case.

2 **Classification Accuracy:** It is the most often used performance statistic for classification algorithms. It may be defined as the proportion of right predictions made to total forecasts made. We may compute it using the confusion matrix and the following formula (1) [66]: -

$$Accuracy=\frac{TP+TN}{TP+FP+FN+TN}$$ (1)

3 **Precision:** The limits of accuracy are addressed by precision. The percentage of accurate positive predictions is known as precision. It is assessed as the proportion of accurate positive forecasts (TruePositive and FalsePositive) to the total number of positive forecasts, following formula (2) [66].

$$Precision=\frac{TP}{TP+FP}$$ (2)

4 **Recall or Sensitivity:** This measure determines the percentage of real positives that were incorrectly detected, much like the Precision metric does. The number of positives properly forecasted as positive or wrongly predicted as negative (true Positive and false negative, respectively) can be used to compute it following formula (3) [66].

$$Recall=\frac{TP}{TP+FN}$$ (3)

5 **F1-Score:** F-score or F1 Score is a metric used to assess the accuracy of a binary classification model’s positive class predictions. Precision and Recall are applied to the calculation. It is a kind of composite score that combines Precision and Recall. As a result, the F1 Score may be derived by taking the harmonic mean of accuracy and recall and assigning equal weight to each. It following formula (4):

$$F1-Score=\frac{2*Precision\times Recall}{Precision+Recall}$$ (4)

6 **Specificity:** In contrast to recall, specificity is the number of negatives returned by our ML model. Using the following formula (5), can readily calculate it using a confusion matrix−

$$Specificity=\frac{TN}{TN+FP}$$ (5)

7 **Loss:** In both training and validation sets, the loss is determined, and its interpretation depends on how well the model performs in each of these two sets. This value represents the total errors created for each instance in a given example’s validation or training sets. After every optimization iteration the model’s loss value reveals how well or poorly it performs. The term “loss” refers to the prediction error of a neural network. The loss function specifies how the loss is computed. Loss is used in the calculation of the gradients. In addition, gradients are employed to change the weights of the neural net as it learns new information.

### 5.4. Results discussion

This section depicts the obtained accuracy and loss value results, followed by data distributions. It also shows the results as in the confusion matrix for classification results, and the ROC curve shows the validation score for this proposed model. The data are now scaled between 0 and 256, although we prefer it to be scaled between 0 and 1. As a result, the data will be compatible with a wide range of deep classification techniques. We also intend to allocate 20% of the dataset for the test.

**Fig 6:**
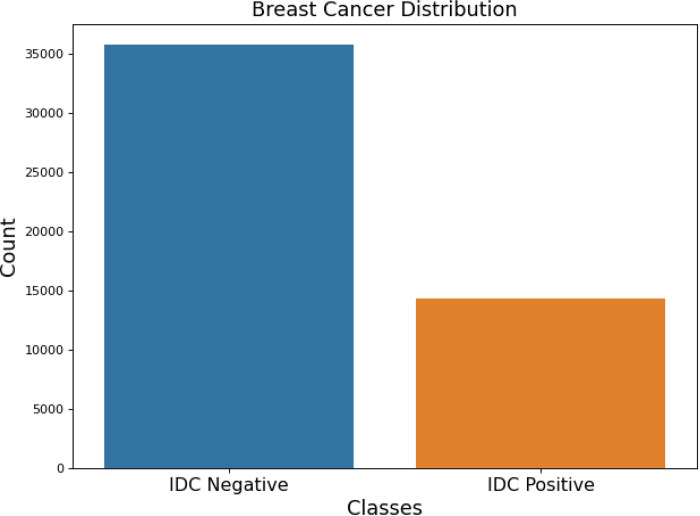
Imbalanced Dataset Distribution.

**Fig 7:**
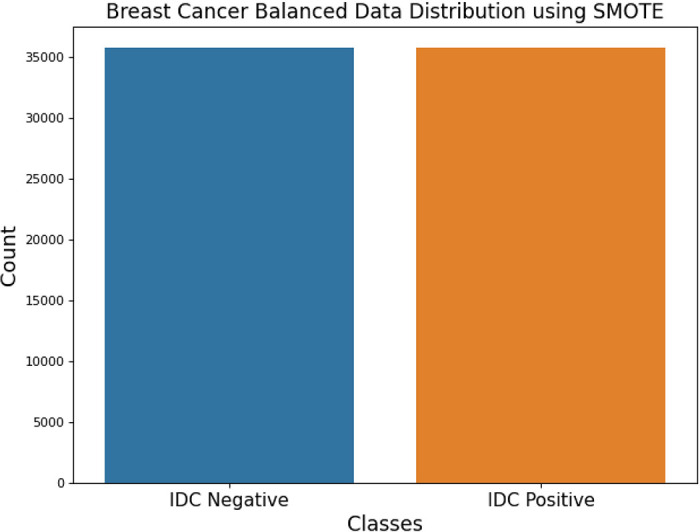
Balanced Dataset Distribution.

This process makes the trained model less susceptible to overfitting, as shown in Fig 6. The breast cancer data are imbalanced, where IDC negative (represented by blue color) has many sample images (35,000). In contrast, the IDC positive class (represented by orange color) has a minimum number of counts of 14,000. Finally, an oversampling method addressed the uneven class sizes shown in Fig 7.

Fig 7 shows the balanced data distribution after applying SMOTE oversampling as a balancing technique. This technique balances both types of classes of IDC that are equally distributed. Both IDC positive and negative have a similar count of approximately 35,000.

**Fig 8:**
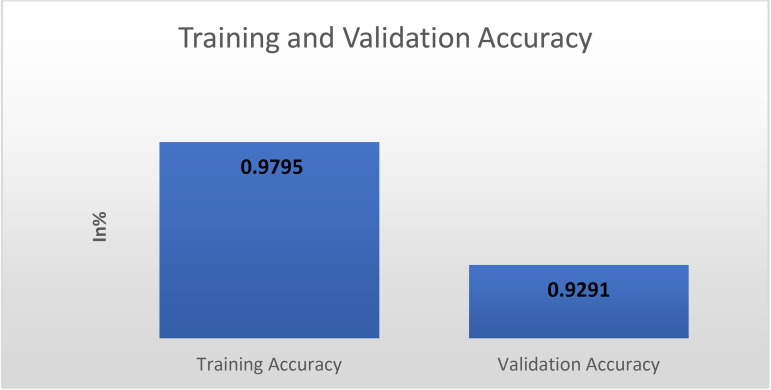
Accuracy Graph.

Fig 8 displays the accuracy result for the proposed model (SMO_CNN). The x-axis represents the SMO_CNN model for measuring the validation and training accuracy over 50 epochs. The y-axis represents the loss or error value achieved by this model. Based on this bar graph, the proposed SMO_CNN model obtained the highest 0.9791% training accuracy and 0.9291% validation loss, respectively.

**Fig 9:**
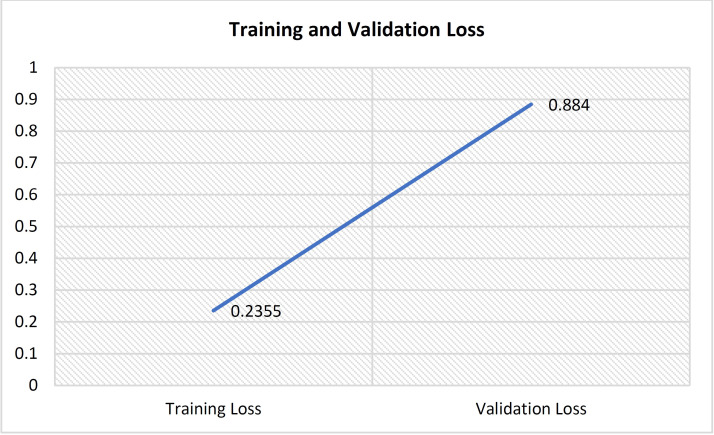
Loss Graph.

Similarly, Fig 9 shows the result of the loss value by using SMO_CNN. The x-axis represents the SMO_CNN model for measuring the training and validation loss. The y-axis represents the loss or error value achieved by this model. Based on this line graph, the proposed SMO_CNN model reduced the loss value in both cases (training loss is 0.23 and validation loss is 0.88) over the 50 epochs.

**Fig 10:**
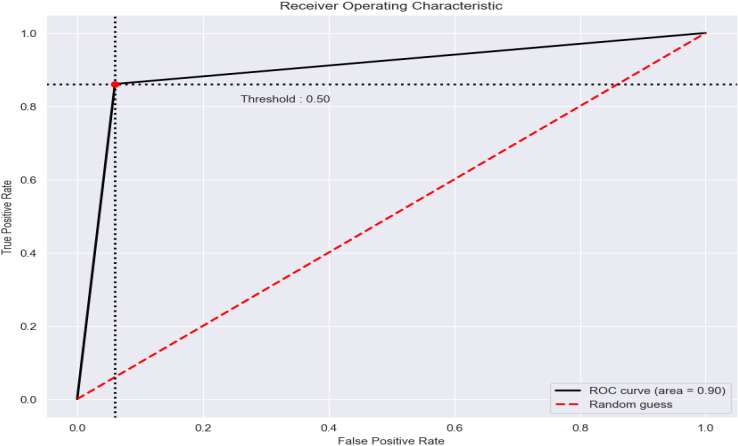
ROC Curve for the Best Model (AUC = 0.92).

The classification model achieved the highest (maximum) validation accuracy of 92.84% during the training phase. Fig 10 depicts the ROC AUC (area under the curve) value for such a model, 0.90. Aside from the average results, the best-case scenario is also important because during the SMO_CNN training, validation accuracy for every epoch may be modified, and the model’s best result may be preserved for use because it is the best scenario.

**Fig 11:**
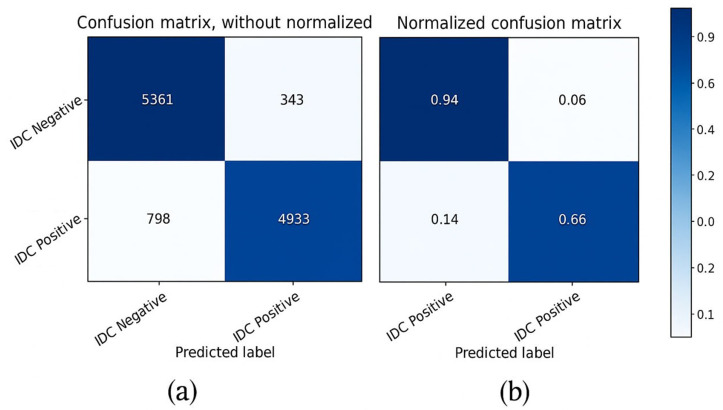
Confusion Metrices: (a) Without normalized (b) Normalized.

The confusion matrix obtained is as follows:

Fig 11 shows the confusion matrix with and without normalization. In circumstances like the one shown in Fig 11a, having a lower FN is preferable to having a lower FP. This preference is attributed to the fact that misdiagnosis of an IDC-negative tumor as an IDC-positive tumor is more dangerous than misdiagnosing an IDC-positive tumor as an IDC-negative tumor because the former will result in the patient receiving a different treatment as a result of the misdiagnosis. In contrast, the final is expected to undergo more tests regardless. The accuracy of 88 percent on the test set shown in Fig 11b demonstrates that our model works well on the test set. We now have a model with a minimum variance due to the confusion matrix, which is another benefit for us.

The following are the possible values of the Confusion matrix for Fig 11a and 11b that might be encountered:

- In Fig 11a, True Negative (TN) = 5361; that is, 5361 data points without normalization from the negative class were properly identified as IDC negative by the model. In Fig 11b, TN = 0.94; the model properly identified 0.94 data points with normalization from the negative class as IDC negative.
- In Fig 11a, True Positive (TP) = 4933; that is, 4933 data points without normalization from the positive class were properly identified as IDC positive by the model. In Fig 11b, TP = 0.86; the model properly identified 0.86 data points with normalization from the positive class as IDC positive.
- In Fig 11a, False Negative (FN) = 798; that is, the model wrongly identified 798 data points without normalization from the negative class as IDC negative. In Fig 11b, FN = 0.14; the model wrongly identified 0.14 data points with normalization from the negative class as IDC negative.
- In Fig 11a, False Positive (FP) = 343; the model properly identified 343 data points without normalization from the positive class. In Fig 11b, FP = 0.06, the model properly identified 0.06 data points with normalization from the positive class as belonging to the IDC positive class.

In this work, we haven’t used k-fold cross-validation to show the different results on different data splitting ratios. Data is splitted into five times with different ratios to obtain these results. Table 6 shows the performance results of different data split ratios based on training, testing, and validation. Here, we have splitted the dataset into five times with random sample selection in terms of training, testing, and validation, i.e., 60:20:20, 75:20:5, 70:20:10, 85:5:10, and 80:10:10, etc. For each sample ratio, we obtained different results of accuracy and loss. The highest results were obtained on the 60%, 20%, and 20% data splitting compared to the other combination ratio.

**Table 6:** Performance Results of Different Data Split Ratios Based on Training, Testing, and Validation.

Data Split Ratio	Training Acc	Testing Loss	VAL Acc	VAL Loss
60:20:20	0.9224	0.3614	0.8694	0.4565
75:20:5	0.9178	0.3798	0.8642	0.3505
70:20:10	0.8966	0.3819	0.8560	0.3666
85:5:10	0.9206	0.3764	0.8622	0.3821
80:10:10	0.9121	0.3092	0.8601	0.4116

Table 7 shows contrast to recall, specificity is the number of negatives returned by our machine learning model. Using the following formula, we can readily calculate it using a confusion matrix. This model obtained the highest 99.20% of training accuracy, 98.84% of validation accuracy, 96.28% of precision, 97.02% of recall, 98.01% of f1-score, 93.98% of sensitivity, and 86.07% of specificity, respectively.

**Table 7:** Performance Results of Proposed SMO_CNN Model.

Model	Training Accuracy	Validation Accuracy	Precision	Recall	F1-Score	Sensitivity	Specificity
SMO_CNN	99.20%	98.84%	96.28%	97.02%	98.01%	93.98%	86.07%

### 5.5. Comparison between proposed and different CNN pre-trained models

**Fig 12:**
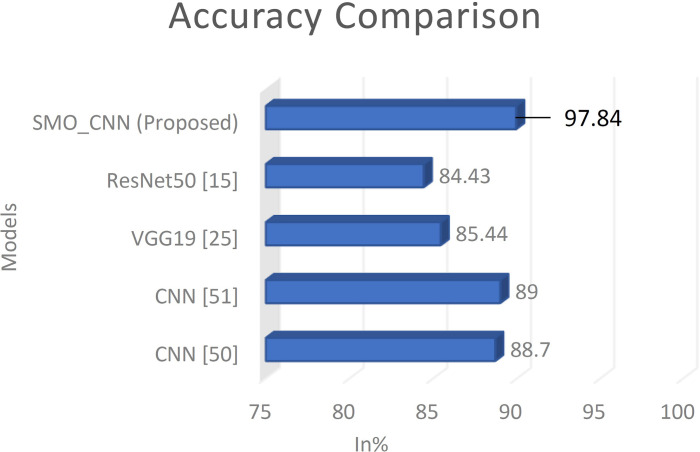
Comparison of Accuracy Metric.

In this section, we contrasted the outcomes of studies performed on the IDC dataset to the evaluation of training accuracy and loss. Comparative experimental results were obtained using the IDC dataset to validate the loss and accurate analysis of the VGG19 ResNet50 and SMO_CNN models. Table 6 compares the performance metrics of the three CNN models, including the existing VGG19 and ResNet50 pre-trained models and the proposed SMO_CNN model. Fig 12 illustrates the bar chart to compare training accuracy (indicated by red color) and validation accuracy (indicated by purple color) for all included deep learning models. The x-axis represents the accuracy value multiplied by 100, and the achieved accuracy is depicted in percentage. The y-axis represents models for training and testing on the IDC dataset.

Table 8 represents the comparative performance results at different optimizers, learning rates, and image sizes. From this comparative analysis, we see that the SGD optimizer achieved minimized accuracy (87.06% training and 88.07% validation) and high training loss (0.339) but the validation loss (0.291) is minimal. While the AdaGrad optimizer performed better than the SGD optimizer in terms of accuracy (89.53% training and 91.15% validation) and training loss (0.254), the validation loss is 0.361. However, the AdaGrad optimizer performs better than SGD but has not performed as well as the Adam optimizer. Adam optimizer achieved high training (94.89%) and validation accuracy (91.32%). It minimized training loss (0.131) compared to both optimizers and minimized validation loss (0.337) compared to the AdaGrad optimizer, but it has a higher validation loss than SGD.

**Table 8:** Comparison Of Performance at Different Optimizers and Learning Rates.

Optimizer	Learning rate	Image size	Training Loss	Training Accuracy (%)	Validation Loss	Validation Accuracy (%)
AdaGrad	0.001	96 × 96 × 3	0.2548	89.53	0.3614	91.15
SGD	0.0001	64 × 64 × 3	0.3393	92.06	0.2913	92.07
Adam	0.001	32 × 32 × 3	0.1314	94.89	0.3374	91.32

Table 9 and Fig 12 show a comparative graph of the accuracy metric for the proposed SMO_CNN model. The y-axis in this graph shows the various models, while the x-axis shows the percentage values of accuracy. Evidently, the suggested SMO_CNN model attained the maximum accuracy of 97.84%.

**Table 9:** Comparative Performance Results between Proposed and Existing Model.

Model	Accuracy
CNN [67]	88.7
CNN [68]	89
VGG19 [31]	85.44
ResNet50 [20]	84.43
SMO_CNN (Proposed)	97.84

## 6. Conclusion

Breast cancer is the leading cause of cancer-related deaths among females. Timely detection and diagnosis are the most effective and efficient methods for managing tumor proliferation. The study included the creation of a specialized CNN architecture using the Synthetic Minority Over-sampling Technique (SMOTE) using Whole Slide Imaging (WSI) pictures from the Invasive Ductal Carcinoma (IDC) dataset. The purpose was to improve the accuracy of breast cancer detection and diagnosis. The performance of the SMO-CNN model was assessed using the IDC dataset. The achieved validation accuracy of 97.91 percent indicates that our proposed CNN model outperforms other models. Based on many trials and comparisons, our innovative approach outperformed the models in this study. The extent to which DL has been successfully applied to a diverse array of practical problems is really remarkable. No additional IDC breast cancer picture collection was used to validate the suggested models. To enhance the efficiency of the models, further feature extraction and fully linked layers may be included. Enhancements may be made to the suggested tailored deep learning model to achieve more precision.

Below are brief synopses of many potential constraints:

- Insufficient training data might hinder the ability of a DL model to effectively apply its knowledge to fresh data or other populations.
- The effectiveness of the model may be compromised by data quality concerns, such as the absence of information or inaccurate labels.
- The model’s ability to apply its knowledge to new data is hindered when overfitting has taken place.

In order to improve the effectiveness of classification, it is necessary to use advanced deep learning techniques and large, diverse datasets in future research. Given its outstanding performance in realistic image processing, particularly in incorporating attention processes into deep learning algorithms, it is worth considering this technique as a prospective strategy to explore. In relation to databases, it would have been preferable to have a more extensive dataset, such as ImageNet, accessible in order to provide a benchmark for the academic community’s research. Concurrently, researchers are endeavoring to extend this technique to include whole-slide images, a more demanding task but one that might result in greater medical practice advantages.

## Supporting information

S1 Data Impressive accuracy data. (XLSX)

## Abbreviations

**CNN**: Convolutional Neural Network
*IDC*: *Invasive Ductal Carcinoma*
BC: Breast Cancer
TILs: Tumor-infiltrating lymphocytes
MRI: Magnetic Resonance Imaging
ML: Machine Learning
DL: Deep learning; adaptive attention U-net, AAU-net
CSAB: Channel self-attention block
BD: Boundary detection
ASSD: Average Symmetric Surface Distance
AUC: Area Under the Curve
BUSIS: Breast ultrasound dataset.
